# The utility of electrodiagnostic inching study and conservative treatment in supracondylar process syndrome: A case report

**DOI:** 10.1097/MD.0000000000020506

**Published:** 2020-05-29

**Authors:** Se-Woong Chun, Seung-Kyu Lim

**Affiliations:** Department of Rehabilitation Medicine, Gyeongsang National University Changwon Hospital, Gyeongsang National University College of Medicine, Changwon, Republic of Korea.

**Keywords:** conservative treatment, electrodiagnostic medicine, inching study, proximal median neuropathy, supracondylar process syndrome

## Abstract

**Rationale:**

Supracondylar process is a rare bony anomaly that can cause neurovascular symptoms. Previous reports on supracondylar process syndrome mostly suspect the condition by physical examination and simple radiograph with little assistance of electrodiagnostic methods and report efficiency of surgical treatment.

**Patients concerns:**

A 45-year-old woman working at an assembly line packing boxes presented with tingling pain at her middle and ring fingers that started 2 months ago. She had positive Tinel sign at the medial side of the distal arm.

**Diagnosis:**

Electrodiagnostic inching study on median nerve was conducted and the conduction velocity at the segment between 3 cm to 5 cm proximal to the elbow crease was decreased to 27m/s. Following imaging studies revealed supracondylar process at 4.2 cm proximal to the medial epicondyle. She was successfully treated with conservative treatment.

**Interventions:**

Oral medications including Non-steroidal anti-inflammatory drug and pregabalin were prescribed along with superficial and deep heat modalities. The extent of manual labor was modified. Additionally, self-massage and stretching/nerve-gliding exercises were delivered.

**Outcomes:**

The symptoms substantially improved and she could sleep without trouble, however, complete resolution was not achieved. After a year, she was nearly symptom-free after changing occupations with only occasional tingling after manual labor of unusual intensity.

**Lessons:**

This case report enlightens the versatility of electrodiagnostic inching study in localizing median neuropathy at the distal arm and the effectiveness of conservative treatment in supracondylar process syndrome.

## 1 Introduction

Supracondylar process of the humerus is a bony protuberance situated on the anteromedial aspect in the distal third of the humerus. It is a rare anatomic variation occurring in 0.4% to 2.7% of the population.^[1,2]^ Most supracondylar processes are asymptomatic,^[3]^ however, it is capable of causing neurovascular symptoms of the median nerve,^[4]^ the brachial artery,^[5]^ and rarely, the ulnar nerve.^[6]^

Symptomatic supracondylar process, namely, supracondylar process syndrome, is very rare and the treatment modalities have not been defined.^[7]^ Previous case reports mostly diagnose the condition based on clinical suspicion encouraged by radiographic evidence and confirm the diagnosis by symptom relief with surgical removal of the bony process. This report presents a case of supracondylar process syndrome diagnosed by electrodiagnostic methods and treated successfully by conservative measures.

## 2 Case report

The patient provided informed consent for the publication of the case report and the accompanying images. The retrospective study design did not require approval of the local ethics committee. A 45-year-old woman visited a secondary hospital complaining of tingling pain at the left middle and ring fingers. The symptom started 2 months ago and was aggravated at and after work and partially relieved by rest. The initially intermittent symptoms became constant and worsened that it interfered with sleep. She was right-handed and worked at an assembly line packing boxes weighing around 5 kg to 6 kg. She had 2 trials of steroid injection at the carpal tunnel at a local clinic. Both injections had partial and temporary effect not lasting more than a week. She was referred to the physiatrist for electrodiagnostic examination to rule out carpal tunnel syndrome.

In the examination room, she had positive Phalen sign in her left with no Tinel sign and mild paresthesia at the volar tip of left index, middle and ring fingers. The sensory and motor function of the ulnar and radial nerves were intact. There was no apparent atrophy nor perceivable weakness of any muscles. During manipulating the left upper extremity to position it for the nerve conduction study, she had an episode of shooting pain starting from the medial arm through the anterior forearm to the middle finger. She acknowledged appended history of mild left medial arm pain just proximal to the elbow that have lasted for several months. Thorough palpation around the area revealed an intense tender point with positive Tinel sign of the median nerve at the left medial distal arm.

**Figure 1:**
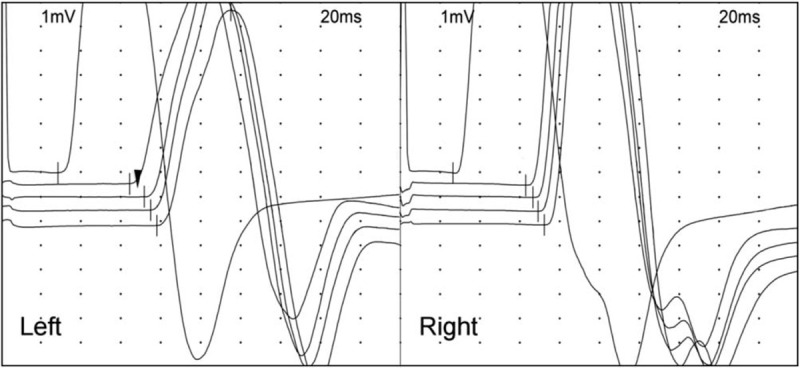
Waveforms of inching study on the median nerve at the distal arm. The compound motor action potential latency of the median nerve stimulated at the wrist, 3 cm, 5 cm, 7 cm, and 9 cm proximal to the elbow crease is marked with a vertical bar. The latency difference between the 2nd and 3rd waveform in the left is prolonged compared to the proximal and contralateral segments (arrowhead).

**Figure 2:**
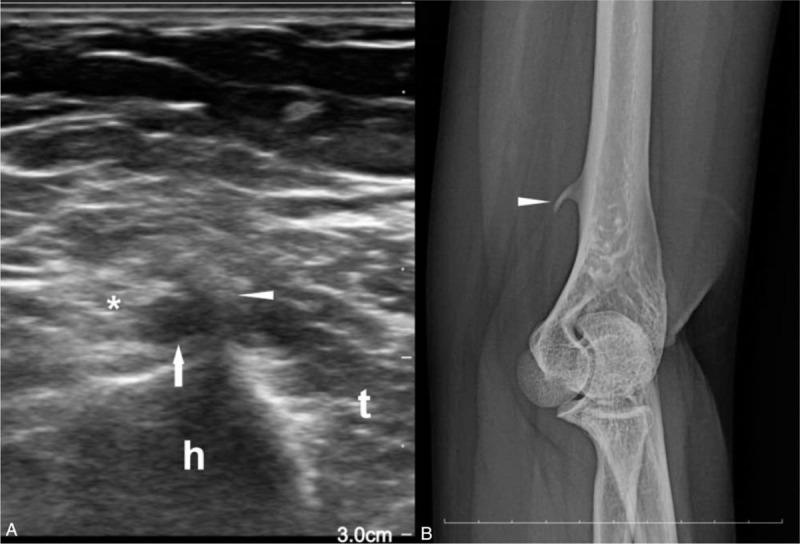
Image of the left distal humerus. A) Ultrasonography. Curved echogenic structure (arrowhead) projecting from the humerus (h) is observed superficial to the median nerve (asterisk) and brachial artery (arrow). t; triceps. B) Simple radiograph. Medial oblique view of the left elbow showed supracondylar process (arrowhead) 4.2 cm proximal to the medial epicondyle.

In the electrodiagnostic examination (Viking Select; Nicolet, San Carlos, CA), distal motor and sensory nerve conduction studies^[8]^ of the median, ulnar and radial nerves showed normal results, including sensitive tests^[9]^ for carpal tunnel syndrome (Table 1). Inching study with recording at abductor pollicis brevis was conducted along the median nerve at the distal arm by short segments of 2 cm. It revealed a segment 3 cm to 5 cm proximal to the elbow crease had decreased conduction velocity of 27m/s in the left side in contrast with the adjacent segments that had conduction velocity of 64m/s (Table 1, Fig. 1). No abnormal findings were seen in the needle electromyography, including the pronator teres, flexor pollicis longus, pronator quadratus, and abductor pollicis brevis. A quick ultrasonographic evaluation (Affinity 70; Philips) was done at site and revealed an echogenic structure superficial to the median and brachial artery (Fig. 2A). Subsequent simple radiograph confirmed a supracondylar process 4.2 cm proximal to the medial epicondyle at the anteromedial aspect of the left humerus (Fig. 2B).

**Table 1:**
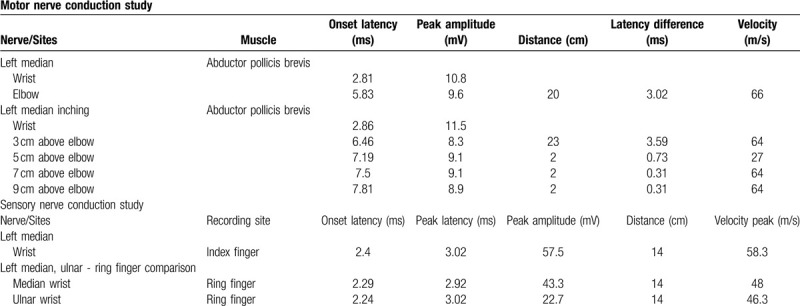
Nerve conduction study.

Oral prednisolone was prescribed by the orthopedic surgeon who referred the patient to the physiatrist and she already have had significant symptom relief before the first visit. Non-steroidal anti-inflammatory drug and pregabalin were prescribed along with superficial and deep heat modalities. She was educated about the disease and advised to modify the extent of manual labor. Additionally, education on self-massage technique lengthwise the median nerve applying shearing force on the surrounding soft tissues and stretching/nerve-gliding exercises were delivered. The symptoms substantially improved and she could sleep without trouble, however, complete resolution was not achieved. She was followed up for 10 months. After a year, she was checked up by telephone and was nearly symptom-free after changing occupations with only occasional tingling after manual labor of unusual intensity. The patient was satisfied by the medication and education delivered at the clinic, however, reviewed that change in occupation was most significant on the improvement.

## 3 Discussion

This case is the first to apply inching study on the median nerve in supracondylar process syndrome and report a discernible abnormal finding that effectively localized the lesion. It is also the first to report successful outcome with conservative treatment for supracondylar process syndrome with naturally developed symptoms.

There are many case reports on the diagnosis and successful treatment of supracondylar process syndrome.^[3,4,6,7,10–22]^ Most cases of supracondylar process syndrome presents with medial elbow pain accompanied with paresthesia in the median nerve distribution. Clinical impression of the condition builds up with associated physical examinations such as tenderness and positive Tinel sign at the medial elbow. The final diagnosis is confirmed by successful clinical outcome after removing the supracondylar process. In the process of the diagnosis of supracondylar process syndrome, the role of eletrodiagnostic medicine seems to be limited in the literature. Most cases reported normal eletrodiagnostic results^[10–15]^ or abnormal findings that did not help localizing the lesion.^[16,19,20]^ Some did not report eletrodiagnostic results^[6,7,18]^ despite electrodiagnostic examination had been available at the time of publication. As a result, clinicians consider electrodiagnostic examination rarely helpful in detecting nerve involvement in supracondylar process syndrome.^[11,19,20]^

Earlier researchers seemed to have been more interested in the electrodiagnostic findings in median neuropathy above the wrist. Roth et al implied that segmental study of the median nerve can be helpful in localizing proximal median neuropathies.^[23]^ Gessini et al and Suranyi reported decreased conduction velocity of 31m/s and 34.5m/s,^[24,25]^ respectively, in the elbow segment in patients with median neuropathy caused by ligament of Struthers which can be similar to supracondylar process syndrome. However, decreased conduction velocity of the elbow segment cannot differentiate supracondylar process syndrome from pronator teres syndrome^[26]^ which is the most common site of median nerve compression above the wrist.^[24]^ Abnormal resting membrane potentials in the pronator teres muscle is the main electrodiagnostic differential point between median nerve compression by supracondylar process and that by pronator teres, however, this finding may also be shown in both conditions.^[26]^

The current case exemplifies the utility of inching study in localizing the lesion in median neuropathy around the elbow. Suranyi unsuccessfully tried the same technique to further localize the lesion after identifying decreased conduction velocity at the elbow segment.^[25]^ The different results between Suryani's report and the current case would be due to the severity of the condition. The patient presented by Suryani had abnormal spontaneous activities in the median innervated muscles which means there was significant axonal involvement and supposedly Wallerian degeneration. At surgery, the median nerve was severely constricted under the ligament of Struthers with proximal swelling and hyperemia. The consecutive orthodromic and antidromic nerve injuries might have delayed the nerve conduction uniformly along the elbow segment. Whereas, the current case had no axonal involvement and had only focal demyelination. In compressive nerve injuries, demyelination generally precedes axon loss.^[26]^ In either cases, inching study is useful in differentiating pronator teres syndrome from more proximal neuropathies because conduction velocity would not be decreased above the elbow in pronator teres syndrome. Especially in milder cases of supracondylar process syndrome where there is no abnormal needle electromyographic findings, inching study would be more helpful in diagnosing and localizing the lesion.

The current case is the first to report successful treatment of supracondylar process of natural symptom generation. There are about fifty case series/reports on supracondylar process syndrome most of which are surgical cases. There seems to be a growing consensus that surgical treatment can be adopted as first-line treatment based on previous articles that report efficiency of surgery after unsatisfying outcome of conservative treatment.^[7,20,27]^ In the literature, there are 5 cases who did not receive surgery. Most reported acceptable therapeutic outcome, however, there are short-comings that make them unappealing as reference of therapy. Surgery was not performed in 2 cases due to patient factor with no report on the final outcome.^[4,11]^ Three were cases with traumatic cause,^[28–30]^ 2 of which had no neurovascular symptoms.^[29,30]^ Acute discomfort after trauma without serious neurovascular compromise would be similar with contusion. Whereas, most cases of supracondylar process syndrome are of insidious onset with a lasting etiology occurring in daily activities with bothersome symptoms that compels the patient to visit the clinician. Although excising the obvious and redundant problematic structure would undoubtedly be an effective treatment, considering the structural cause is a pre-existing condition, modifying the extent of upper extremity utilization can also be effective.

There is a controversy in this case whether the symptom was caused by the supracondylar process or the carpal tunnel. There are previous articles that raise similar issue.^[10,21]^ Patients with supracondylar process syndrome often have carpal tunnel release before correct diagnosis^[10,21]^ and may concomitantly have physical findings^[10,19]^ or electrodiagnostic findings^[10,19]^ implicating carpal tunnel syndrome. Serial constraints of a nerve fiber have synergistic effect in compromising the axoplasmic flow.^[31]^ The exact region of median nerve compression cannot be unequivocally determined in the current case. The authors consider both contributed to the patient's chief complaint with the supracondylar process being the precondition. The chief complaint was confined to distal to the wrist and Phalen sign was positive. These facts imply that the chief complaint can be attributed to compression at the carpal tunnel to some extent. However, there was no electrodiagnostic abnormality at the carpal tunnel while there was definite focal demyelination around the supracondylar process. The electrodiagnostic findings along with the ancillary symptoms above the wrist favor the dominant share of the supracondylar process in the generation of the symptoms.

## 4 Conclusion

This case exemplifies the versatility of electrodiagnostic medicine via successful localization of the supracondylar process with inching study on the median nerve. Opposed to suggested in literature, electrodiagnostic medicine can promote differentiating and localizing median neuropathy in supracondylar process syndrome. And supracondylar process syndrome can be treated successfully by conservative measures.

## Acknowledgments

We would like to thank the patient's agreement for the publication and HARRISCO (en.harrisco.net) for English language editing.

## Author contributions

**Conceptualization:** Se-Woong Chun

**Resources:** Se-Woong Chun

**Supervision:** Seung-Kyu Lim

**Writing – original draft:** Se-Woong Chun

**Writing – review and editing:** Seung-Kyu Lim
